# Visualized Multiprobe Electrical Impedance Measurements with STM Tips Using Shear Force Feedback Control

**DOI:** 10.3390/s16060757

**Published:** 2016-05-25

**Authors:** Luis Botaya, Xavier Coromina, Josep Samitier, Manel Puig-Vidal, Jorge Otero

**Affiliations:** Department of Electronics, Universitat de Barcelona, Barcelona 08028, Spain; lbotaya@el.ub.edu (L.B.); xcoromina@el.ub.edu (X.C.); jsamitier@ub.edu (J.S.)

**Keywords:** scanning tunneling microscope (STM) tip, scanning probe microscopy, multiprobe SPM, quartz tuning forks, impedance measurement

## Abstract

Here we devise a multiprobe electrical measurement system based on quartz tuning forks (QTFs) and metallic tips capable of having full 3D control over the position of the probes. The system is based on the use of bent tungsten tips that are placed in mechanical contact (glue-free solution) with a QTF sensor. Shear forces acting in the probe are measured to control the tip-sample distance in the *Z* direction. Moreover, the tilting of the tip allows the visualization of the experiment under the optical microscope, allowing the coordination of the probes in *X* and *Y* directions. Meanwhile, the metallic tips are connected to a current–voltage amplifier circuit to measure the currents and thus the impedance of the studied samples. We discuss here the different aspects that must be addressed when conducting these multiprobe experiments, such as the amplitude of oscillation, shear force distance control, and wire tilting. Different results obtained in the measurement of calibration samples and microparticles are presented. They demonstrate the feasibility of the system to measure the impedance of the samples with a full 3D control on the position of the nanotips.

## 1. Introduction

Since the invention of the scanning tunneling microscope (STM) in 1981 by Binning and Rohrer [1], the investigation of the local electrical properties of a wide range of samples at the nanoscale has been widely reported [2,3]. The main idea is to measure the electrical interactions between a sharp metallic tip (nanometric tip radius) and the surface to determine the local properties of the sample of study. The original microscopy technique was developed by using the exponential decay of the tunneling current with the tip-sample distance. Controlling this current, it is possible to keep the tip-sample distance constant while scanning the sample to acquire the surface topography. The limitation of working with conducting samples was overcome with the development of the atomic force microscope [4], where the nanometric tip is placed on a microfabricated cantilever. Then, the bending of this cantilever is measured to determine the atomic forces between the tip and the sample surface (which depend on the tip-sample distance and have an exponential decay as well [5]), thus allowing for the reconstruction of the surface topography if the sample is scanned while keeping the force interaction constant. Evolved from these, several techniques have appeared over the last several decades to measure the nanoelectrical properties of the sample, where we can clearly identify two different groups: wire-based and cantilever-based techniques. Wire-based techniques (STM [6], electrochemical STM [7], scanning electrochemical microscopy (SECM) [8], scanning ion-conductance microscopy (SICM) [9], *etc.*) are limited in that the tip-sample distance is controlled by electrical measurement meanings, thus introducing artifacts due to the coupling between the topography and electrical properties of the surface. On the other hand, cantilever-based techniques (conductive atomic force microscopy (C-AFM) [10], Kelvin probe microscopy (KPM) [11], electrostatic force microscopy (EFM) [12], *etc.*) overcome the previous limitation by measuring the interaction forces (and then the topography) independently from the electrical signals; nevertheless, the electrostatic forces that appear between the tip and the sample at nanometric distances make the cantilever bend and collapse towards the sample surface; thus, the techniques are limited to larger tip-sample distances or contact-mode measurements. Simultaneous force and conductance measurements have been taken with cantilevered tips taking into account those considerations [13]. Further, for working in liquid environments, wires are easier to isolate electrically than are microfabricated cantilevers. Finally, both solutions (wire- and cantilever-based) measure the tip-sample electrical properties in *Z* direction, but they cannot determine the *X* and *Y* components (two probes would be needed for that).

Putting it all together, a system which overcomes the limitations of both solutions would be desirable. In this manuscript, we present a system where two sharp wires are used for the measurement by controlling the tip-sample distance independently from the electrical measurements. This is accomplished by mechanically coupling the wires to quartz tuning fork (QTF) sensors to measure the shear force between the tip and the sample. The two sensors are constantly visualized by an optical microscope and can be positioned relatively between them with submicron accuracy; in this way, 3D electrical measurements can be made (in *X*, *Y*, and *Z* directions), together with differential measurements, which ease the data analysis by compensating parasitic effects (mainly the parasitic currents which appear due to the wires and electrical connections of the sample). Additionally, it opens the door for further applications where the optical images can give important information [14]. The use of wires instead of cantilevers also eases working in liquid media by isolating the wires with some of the well-known techniques used in electrochemical scanning tunneling microscopy (EC-STM) [15,16].

## 2. Shear Force Control on Metallic Sharp Probes

In recent years, quartz tuning forks have been used as nanoscopy sensors due to their advantages under certain conditions over standard microcantilever sensors [17,18]. Some of the disadvantages of these QTF sensors are being overcome, such as the lack of a confident value for the spring constant [19] and the difficulty in making the tip radius comparable in size with the ones in commercial probes [20]; therefore, in the present work, QTF sensors were used to control the tip-sample distance. In this way, the shift in the frequency of oscillation of the QTF and the electric current flowing through the electrode are measured independently so that the former can be used to maintain the tip-sample distance constant and the latter to measure the impedance of the sample. The QTF is electrically excited, instead of mechanically, which enables a better quantitative control of the response of the system. The tip is not glued onto the QTF, but the two are in tight mechanical contact, which allows the use of relatively long metallic tips.

QTF devices are mostly used in electronics for precise oscillation circuits. The resonance frequency depends on the effective mass spring constant of the device [21]. We used commercial QTFs encapsulated in vacuum conditions (with a resonance frequency of 32,768 Hz). To use these QTFs as distance nanosensors, they must be removed from their metallic capsules, and the wire must be placed in mechanical contact with one of the prongs of the resonator, thus producing a variation in the resonant frequency of the QTF. The nanosensor is oscillated parallel to the sample surface. When a shear force appears, there is a shift in the resonant frequency of the nano-tool. Control of this shift in the resonance frequency allows us to keep the tip-sample force constant and, hence, the tip at a very short distance from the surface of the sample. It should be noted, however, that metallic tips are heavier than fiber tips (the most common kind of tip used in QTF sensors for scanning probe microscopies). When a metallic tip is glued to a QTF prong, its weight produces resonating modes that make it extremely difficult to use as a nanosensor. We have avoided these problems by fixing the tip to the QTF prong without glue.

## 3. Multiprobe Experimental Setup

**Figure 1:**
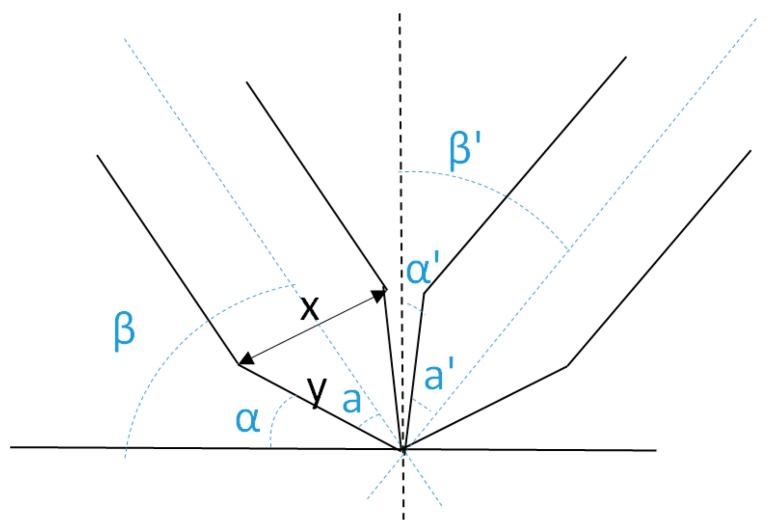
Schematic representation of the geometries of the two tips. The wires should be tilted at a minimum angle *β* from the horizontal axis to ensure that the tip end interacts with the sample. Additionally, the wires should be tilted a minimum angle *β*’ from the vertical axes to ensure the tip visibility from the optical microscope.

In order to work cooperatively under the optical microscope, the tungsten wires needed to be tilted. Some considerations were taken into account on that aspect. As can be seen in Figure 1, the wires needed to be tilted at a minimum *β* angle from the horizontal axis to avoid touching the surface with the cone. Furthermore, they were tilted at a minimum angle *β*’ from the vertical axis to make the tip end visible through the upright optical microscope. If *a* is the cone angle of the tip, the minimum tilt angle is *a*, and the maximum tilt angle is 90°-*a*. The metallic tips selected were commercial tungsten STM tips (TT-ECM10, from Bruker AFM probes), which had a cone angle of 32° ± 6° (measured from 10 different probes); therefore, the wires should be tilted an angle between 38° and 52° from the horizontal axis.

**Figure 2:**
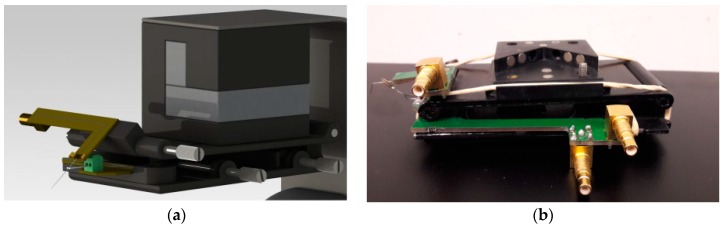
(**a**) 3D CAD design of the head; (**b**) Photograph of the designed head for the multitip system. The wire and the QTF can be positioned independently and precisely by using micrometric screws. Rubber strips are used to maintain the mechanical contact between the wire and the QTF without the need of any glue.

With the visibility from the top, it is possible to precisely position and coordinate the tips in *X* and *Y* directions using the optical microscope information throughout the strategies and control algorithms previously developed in [22]. To control the tip-sample distance and thus the *Z*-axis of the measurement system, QTF sensors were used (AB38T model, Abracon Corp., Irvine, CA, USA). A common way to use these sensors for tip-sample distance control is to glue the tip to them with an epoxy. However, in our application, larger wires than usual were needed, so the added mass to the QTF resonator is heavier than usual. Therefore, it is very difficult to drive electrically the sensor with this huge added mass because the electrical energy, which can be given to the device without damaging it, is very low. On the other hand, electrical excitation of the device was desirable because it simplifies the design, and it allows for the quantification of the measurements (when the QTF is driven mechanically by using a dither piezo, the mechanical coupling between the dither and the QTF should be known for quantification). Following the idea presented in [23] for using a glue-free solution for developing the distance sensor based on optical fibers, authors presented a glue-free solution for metallic tips in a previous work [24]. Briefly, the metallic tip was placed in mechanical contact with the QTF resonator by using a specifically designed holder with a 3D micropositioning system. In that way, the best contact point was found experimentally by looking at the frequency response and then maintained by using a spring-based system. The designed head was adapted so that working with tilted wires in multitip applications was possible, as shown in Figure 2.

**Figure 3:**
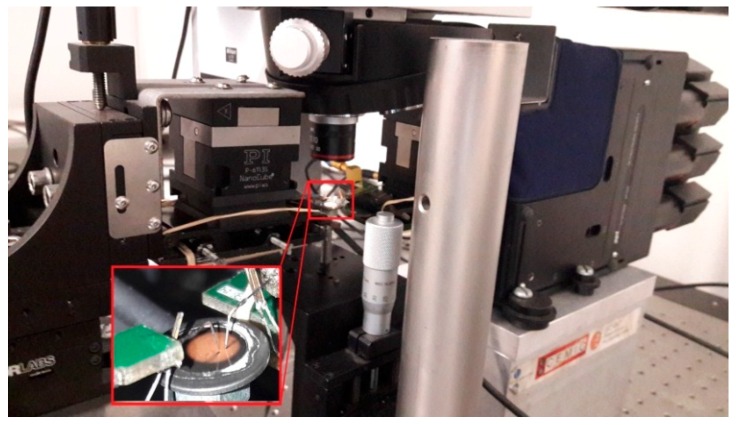
The nanopositioning station with the end-effectors integrated. Microstepper motors are used for coarse positioning and piezoelectric actuators for fine positioning. The sample and the end-effectors are visualized by an upright optical microscope.

Heads with the integrated tungsten wires and QTF sensors were mounted into a micro- and nanopositioning system under an upright optical microscope (Nikkon, custom made development with interchangeable objectives from 5× to 50× magnification), as shown in Figure 3. Macro- and micropositioning of the end-effectors were accomplished with microstepper motors (THORLABS MT3 and APT604 stages) with a resolution of 40 nm and a travel range of 12 mm. Nanopositioning of the tips was accomplished by two piezoelectric positioning systems (PI Nanocube) with a resolution of 1 nm and a travel range of 100 μm. A digital controller (Dulcinea controller from Nanotec Electrónica) with 4 PI controllers, 2 lock-in amplifiers, and 16 analog I/0 channels was used to drive the actuators, measure the sensors, and execute the control algorithms.

**Figure 4:**
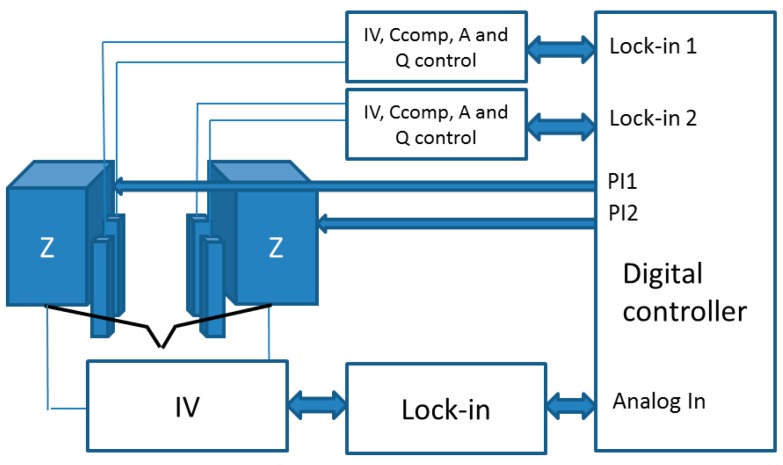
Scheme of the electronics and control system. The shear force between the tips and the surface are measured through custom-designed electronics, and the digital controller uses them to control the tip-sample distance (PI control) by adjusting the *Z*-axis of the piezoelectric actuators. The impedance between the two tips is measured independently by an I–V and a lock-in amplifier.

For the measurement of the shear force in the QTF sensors, a custom-made electronics [25] was used together with the integrated lock-in amplifiers in the digital controller. Briefly, the electronics integrate the parasitic capacitor compensation, which allows for control of the amplitude of vibration and the quality factor of the sensors independently. For the impedance measurement, a custom I–V converter was developed based on the AD549JH amplifier (Analog Devices, Norwood, MA, USA), and a lock-in amplifier (Anfatec eLockIn204/2) was used. A complete scheme of the whole electronic system is presented in Figure 4.

## 4. Results and Discussion

All the experiments presented in this work were carried out in ambient conditions, with an excitation frequency of 5 kHz and using a fixed 10^7^ gain for the I–V converter. Tungsten wires were tilted at approximately 45 degrees with respect to the *X* plane.

The experiments consisted of measuring the impedance between the two probes when they were placed at nanometric distances from the sample surface (distances were the sample impedance dominates, as discussed in the next section). Complex impedance has contributions from resistive, capacitive, and inductive contributions. For the calibration experiments, purely resistive or capacitive samples were used.

### 4.1. Calibration Experiments

**Figure 5:**
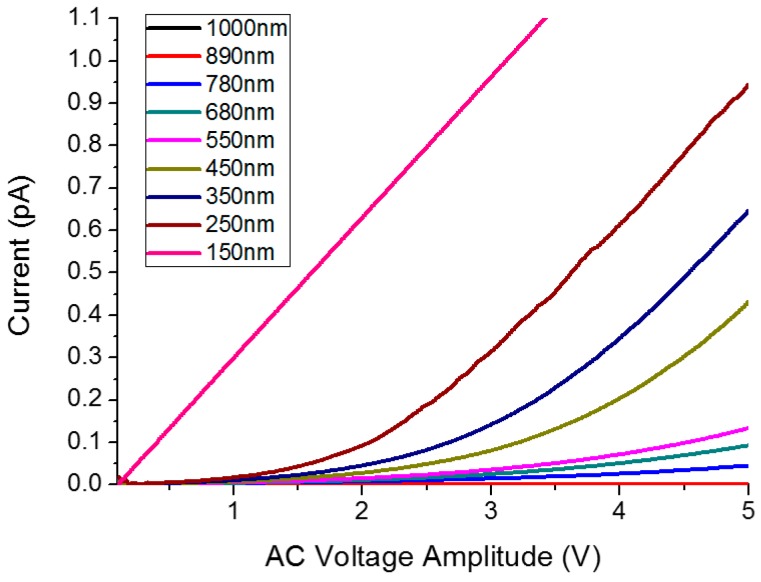
Series of I–V curves over the resistor sample recorded while changing the tip-sample distance. The non-linear behavior due to the tip-sample capacitance is observed for distances greater than 150 nm.

The first experiment to test the ability of the system to measure the sample’s impedance was to determine the optimal working setpoint (tip-sample distance). When the metallic tip approaches the sample, the impedance between the tip and the sample (which depends on the tip-sample distance and the dielectric constant of the medium in between) increases and can be measured. This impedance has a mainly non-linear capacitive behavior [26]. To determine the adequate tip-sample distance where the sample impedance became dominant, a series of I–V curves were recorded while the tip was moved toward the sample. The tip-sample distance was determined from an amplitude *vs.* Z-curve made prior to the measurements (the tip is moved toward the sample surface while recording the changes in the amplitude of oscillation). In this case, the sample was a gold trace connected to a resistor. Figure 5 shows how the non-linear behavior (due to the tip-sample capacitance) was progressively reduced by moving the tip toward the sample up to a certain distance (150 nm), where only the linear (resistive) behavior of the sample was observed. This distance was used for the subsequent measurements in this work.

**Figure 6:**
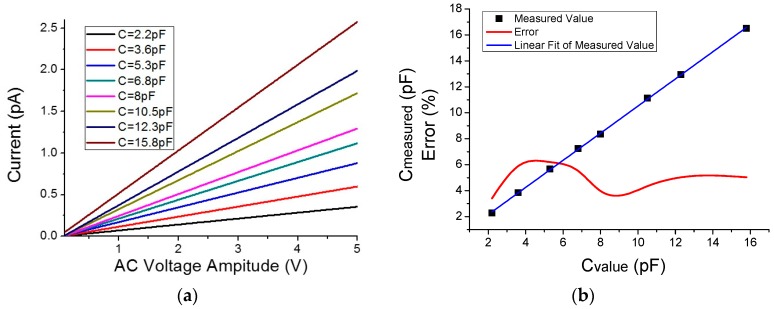
Capacitors measurements. (**a**) Curves obtained at 5 kHz over known-value capacitors by varying the voltage amplitude and measuring the current. (**b**) Error in the measurements. The I–V curves display the expected linear behavior. The measured values are in great accordance with nominal values, with errors lower than 6%. Linear fit equation is 0.07 + 1.045x with adjusted R^2^ of 0.9997.

Once the minimum tip-sample distance was determined, a series of purely capacitive samples were measured (gold traces connected to discrete capacitors of a known value). The tip-sample distance was maintained below 150 nm so that the sample capacitance (linear) dominated over the tip-sample capacitance (non-linear). The amplitude of the signal at 5 kHz was a sweep between −5 V and 5 V. Results for the positive sweep (negative amplitudes produced symmetrical results) are shown in Figure 6.

Capacitors were measured precisely prior to the measurements with an impedance analyzer, which gave the values used as “reference” (2.2 pF, 3.6 pF, 5.3 pF, 6.8 pF, 8 pF, 10.5 pF, 12.3 pF, and 15.8 pF). Thus, results obtained with the developed system displayed a maximum difference of 6% with these previous “reference” values, so we think that the results demonstrated that the system is capable of measuring a purely capacitive sample with ample accuracy.

### 4.2. Microparticle Impedance Measurement

**Figure 7:**
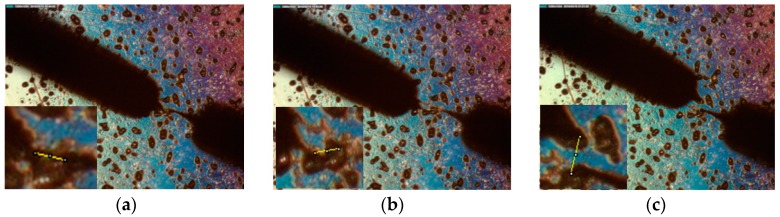
Microparticle measurement experiment as visualized with the optical microscope at distances of 22 μm, 45 μm, and 56 μm between the tips. (**a**) Both tips over the same microparticle; (**b**) tips over microparticles in contact with each other; (**c**) tips over microparticles that are not in contact with each other.

For the sample preparation, aluminum microparticles (of sizes ranging from 10 to 100 μm, purchased from Pomenton Inc., Maerne, Italy) where diluted in ethanol. A drop of the solution was deposited onto freshly cleaved mica and allowed to evaporate naturally. Then, the two tungsten wires were place over the sample. The left tip was placed over a microparticle, and the right tip was then placed at three different positions: over the same microparticle (Figure 7a), over a different microparticle in contact with the first one (Figure 7b), and over a different microparticle that was not in contact with the first one (Figure 7c).

**Figure 8:**
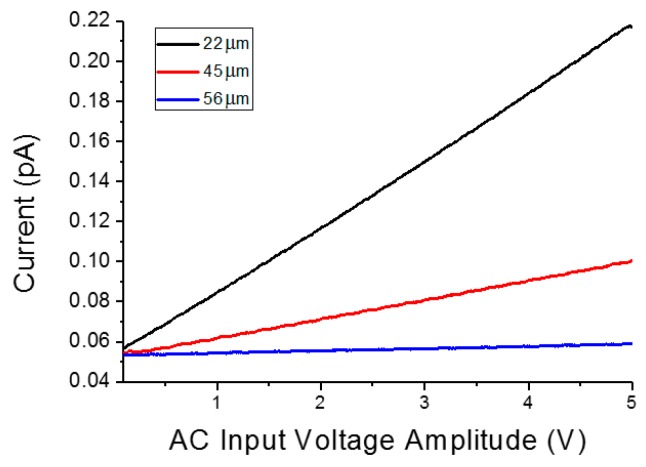
Current–voltage curves with one tip over a microparticle and the second tip at different distances. As expected, the impedance increased with the distance between the tips, and as the tips contacted the same microparticle (a distance of 22 μm), two microparticles in contact with each other, (a distance of 45 μm) and two microparticles not in contact with each other (a distance 56 of μm).

Current–voltage curves on the three measurements are shown in Figure 8. When both tips are over the same microparticle, an impedance of 30.4 MΩ is measured, which one can assume is the impedance of a single microparticle. When two microparticles in contact are measured, the impedance measured is higher (104.1 MΩ) than it is for a single microparticle; impedance is more than double due to the effects of the pseudo-spherical geometry of the particles and the non-ideal ohmic contact between them. Finally, when the tips are placed over two different particles that are not in contact with each other, the impedance increases to almost one order of magnitude (876.8 MΩ); thus, the capacitor formed by the two particles and the dielectric (mica and air) between them is measured.

## 5. Conclusions

Here we report the architecture, design, and validation of a novel, customized multitip system based on a QTF with a glue-free metallic tip working in shear mode for impedance measurements. Both tips work simultaneously as force and current nanosensors. The considerations on the wire tilting and tip-sample distance have been presented, as well as calibration experiments over purely resistive and capacitive samples. Finally, an experiment with aluminum microparticles has been presented.

The system presented here overcomes most of the limitations of the existing solutions offered in the literature. The tip-sample distance can be controlled independently from the measured current, and differential measurements can be taken due to the two-sensors configuration. Additionally, the use of wires instead of cantilevers, which are easy to electrically isolate, opens the door for future applications in liquid media.

## References

[1] Binning G., Rohrer H. (1986). Scanning tunneling microscopy. IBM J. Res..

[2] Mathwig K., Aartsma T., Canters G., Lemay S. (2014). Nanoscale Methods for Single-Molecule Electrochemistry. Ann. Rev. Anal. Chem..

[3] Raigoza A., Dugger J., Webb L. (2014). Review: Recent Advances and Current Challenges in Scanning Probe Microscopy of Biomolecular Surfaces and Interfaces. ACS Appl. Mater. Interfaces.

[4] Binning G., Quate C., Gerber C. (1986). Atomic Force Microscope. Phys. Rev. Lett..

[5] Hofer W., Fisher A. (2003). Signature of a Chemical Bonde in the Conductance between Two Metal Surfaces. Phys. Rev. Lett..

[6] Bowker M., Davies P. (2010). Scanning Tunneling Microscopy in Surface Science, Nanoscience and Catalysis.

[7] Itaya K., Tomita E. (1988). Scanning tunneling microscope for electrochemistry—A new concept for the *in situ* scanning tunneling microscope in electrolyte solutions. Surf. Sci..

[8] Bard A., Fan K. (1989). Scanning Electrochemical Microscopy. An introduction and Principles. Anal. Chem..

[9] Hansma P., Drake B., Marti O., Gould S., Prater C. (1989). The scanning ion-conductance microscope. Science.

[10] Houze F., Meyer R., Schneegans O., Boyer L. (1996). Imaging the local properties of metal surfaces by atomic force microscopy with conducting probes. Appl. Phys. Lett..

[11] Nonnenmacher M., O’Boyle M., Wickramasinghe H. (1991). Kelvin probe force microscopy. Appl. Phys. Lett..

[12] Law B., Rieutord F. (2002). Electrostatic forces in atomic forces microscopy. Phys. Rev. B.

[13] Sawada D., Sugimoto Y., Morita K., Abe M., Morita S. (2009). Simultaneous measurement of force and tunneling current at room temperature. Appl. Phys. Lett..

[14] Wei Y., Wu C., Wang W. (2016). Shape Reconstruction Based on New Blurring Model at the Micro/Nanometer Scale. Sensors.

[15] Penner R., Heben M., Longin T., Lewis N. (1990). Fabrication and Use of Nanometer-Sized Electrodes in Electrochemistry. Science.

[16] Guell A., Diez-Perez I., Gorostiza P., Sanz F. (2004). Preparation of reliable probes for electrochemical tunneling spectroscopy. Anal. Chem..

[17] Otero J., Gonzalez L., Puig-Vidal M. (2012). Nanocharacterization of Soft Biological Samples in Shear Mode with Quartz Tuning Fork Probes. Sensors.

[18] Wu Z., Guo T., Tao R., Liu L., Chen J., Fu X., Hu X. (2015). A Unique Self-Sensing, Self-Actuating AFM Probe at Higher Eigenmodes. Sensors.

[19] Gonzalez L., Oria R., Botaya L., Puig-Vidal M., Otero J. (2015). Determination of the static spring constant of electrically-driven quartz tuning forks with two freely oscillating prongs. Nanotechnology.

[20] Gonzalez L., Martinez-Martin D., Otero J., de Pablo P., Puig-Vidal M., Gomez-Herrero J. (2015). Improving the Lateral Resolution of Quartz Tuning Fork-Based Sensors in Liquid by Integrating Commercial AFM Tips into the Fiber End. Sensors.

[21] Castellanos A., Agraït N., Rubio-Bollinger G. (2009). Dynamics of quartz tuning fork sensor used in scanning probe microscopy. Nanotechnology.

[22] Otero J., Gonzalez L., Cabezas G., Puig-Vidal M. (2013). Multitool Platform for Morphology and Nanomechanical Characterization of Biological Samples with Coordinated Self-Sensing Probes. IEEE/ASME Trans. Mechatron..

[23] Mühlschlegel P., Toquant J., Pohl D., Hecht B. (2006). Glue-free tuning fork shear-force microscope. Rev. Sci. Instr..

[24] Botaya L., Otero J., Gonzalez L., Coromina X., Gomila G., Puig-Vidal M. (2015). Quartz tuning fork-based conductive atomic force microscope with glue-free solid metallic tips. Sens. Actuators A Phys..

[25] Gonzalez L., Otero J., Cabezas G., Puig-Vidal M. (2012). Electronic Driver with Amplitude and Quality Factor Control to Adjust the Response of Quartz Tuning fork Sensors in Atomic Force Microscopy Applications. Sens. Actuator A Phys..

[26] Fumagalli L., Ferrari G., Sampietro M., Gomila G. (2009). Quantitative nanoscale dielectric constant measurement of thin films by DC electrostatic force microscopy. Nano Lett..

